# Automated Phenotype Recognition for Zebrafish Embryo Based *In Vivo* High Throughput Toxicity Screening of Engineered Nano-Materials

**DOI:** 10.1371/journal.pone.0035014

**Published:** 2012-04-10

**Authors:** Rong Liu, Sijie Lin, Robert Rallo, Yan Zhao, Robert Damoiseaux, Tian Xia, Shuo Lin, Andre Nel, Yoram Cohen

**Affiliations:** 1 Center for the Environmental Implications of Nanotechnology, California Nanosystems Institute, University of California Los Angeles, Los Angeles, California, United States of America; 2 Department of Chemical and Biomolecular Engineering, University of California Los Angeles, Los Angeles, California, United States of America; 3 Departament d'Enginyeria Informatica i Matematiques, Universitat Rovira i Virgili, Tarragona, Catalunya, Spain; 4 Department of Molecular, Cell and Developmental Biology, University of California Los Angeles, Los Angeles, California, United States of America; 5 Molecular Shared Screening Resources, University of California Los Angeles, Los Angeles, California, United States of America; 6 Department of Medicine - Division of NanoMedicine, University of California Los Angeles, Los Angeles, California, United States of America; Universitat Rovira i Virgili, Spain

## Abstract

A phenotype recognition model was developed for high throughput screening (HTS) of engineered Nano-Materials (eNMs) toxicity using zebrafish embryo developmental response classified, from automatically captured images and without manual manipulation of zebrafish positioning, by three basic phenotypes (i.e., hatched, unhatched, and dead). The recognition model was built with a set of vectorial descriptors providing image color and texture information. The best performing model was attained with three image descriptors (color histogram, representative color, and color layout) identified as most suitable from an initial pool of six descriptors. This model had an average recognition accuracy of 97.40±0.95% in a 10-fold cross-validation and 93.75% in a stress test of low quality zebrafish images. The present work has shown that a phenotyping model can be developed with accurate recognition ability suitable for zebrafish-based HTS assays. Although the present methodology was successfully demonstrated for only three basic zebrafish embryonic phenotypes, it can be readily adapted to incorporate more subtle phenotypes.

## Introduction

In many modern industrial products and processes, materials of nano-size are increasingly utilized as common elements primarily due to their novel properties that arise at the nano-scale [1]. Engineered Nano-Materials (eNMs) are estimated to be components of more than 1,000 commercial products [2], and this number is expected to grow significantly in the forthcoming years. As a result, there is increased public concern regarding the potential for adverse environmental and health impacts associated with eNMs throughout their lifecycle [3]. Given the large number of existing and expected eNMs types, considerable effort has been devoted to developing high throughput screening (HTS) methods for eNM toxicity [4]–[7]. Information regarding eNM toxicity via HTS studies provides fundamental building blocks necessary for the development of risk assessment strategies and to assist the development of environmental and health regulatory policies [6].

HTS toxicity studies of eNMs are accomplished primarily via *in vitro* screening [8]. *In vitro* HTS toxicity screening methods, however, often lack the desired predictability for eNM toxicological assessment in whole organisms because of the increased complexity of an *in vivo* biological environment, including the environmental media, in which the analysis is being performed [8]. In contrast, *in vivo* animal studies (using zebrafish, mice, guinea pigs, etc.), although more expensive, complex, and laborious [9]–[11] relative to cellular HTS toxicity screening, are typically considered as more definitive regarding toxicity assessment [12]. Recently, efforts to bridge *in vitro* (e.g., using cell cultures) with *in vivo* eNM toxicological assessment have focused on zebrafish (*Danio rerio*) [13]–[15] as a model organism for *in vivo* toxicity and teratogenicity screening [16]–[22]. In this regard, it is noted that the National Institute of Environmental Health Sciences (NIEHS) in the United States and the Institute for Environment and Sustainability (IES) in Europe both support the use of zebrafish as a basic model organism for the assessment of environmental toxicity [23], [24]. Furthermore, the National Institutes of Health (NIH) recognizes the zebrafish as an alternative model for exploring human disease, development, and physiology [23], [24].

The major advantages of using zebrafish for HTS toxicity studies include: (a) large number of embryos can be obtained at low cost, (b) zebrafish embryos undergo rapid development from eggs to larvae in three days, (c) zebrafish embryos and larvae can be kept alive in micro-plates for days, and (d) zebrafish embryos and larvae are close to being optically transparent [25], [26]. As the application of zebrafish-based toxicity assays expands in HTS studies, researchers will be confronted with the challenge of efficiently resolving/extracting the latent semantics (e.g., phenotypic maldevelopment of zebrafish embryos in exposure to eNMs) embedded in the potential large number of images being generated in a single experiment [25]. In order to isolate and quantify the image based data, the majority of the published studies on zebrafish high throughput screening have resorted primarily to fluorescence-based microscopy using specifically developed transgenic zebrafish lines (e.g., Tg(fli1:EGFP)) [27]–[32]. For example, through the use of fluorescence intensity and distribution, an automated high-throughput mapping of promoter-enhancer interactions in zebrafish embryos was recently developed [29]. The reporter gene expression in the embryos was registered (i.e., categorized) to eight domains (yolk ball, eye, skin, brain domain, midbrain-hindbrain boundary, heart, spinal cord, and notochord) via an image-based method exhibiting an average registration accuracy of 86%. Another recent study also adopted fluorescence-based microscopy and employed cognition network technology (an object-oriented image analysis method that emulates cognitive processes in the human mind) to quantify intersegmental blood vessel development from images of zebrafish embryos with an error rate of 4.5% [31]. Although the use of fluorescence-based microscopy can improve image analysis of HTS zebrafish screening, it requires upfront construction of transgenic zebrafish lines. On the other hand, for non-fluorescence based HTS, the usual grayscale image analysis is significantly more challenging. Recently, a bright-field (grayscale) zebrafish image analysis algorithm, based on a heuristic approach, was proposed that detects and segments a region enclosing an area surrounding the pigments [25] (a.k.a., the Region of Interest, ROI). The pigmentation in the ROI could reflect the response of the zebrafish embryos to various environmental cues [25]. In the above approach, the ROI was detected from images acquired from 24-well plates with the help of a priori anatomical information of zebrafish embryos. The approach was tested using 18 images of zebrafish embryos treated with dimethyl sulfoxide and gamma secretase inhibitor (GSI-18) and resulted in false positive and negative identification rates (compared to a manual analysis) of 28.6% and 37.5%, respectively. The authors indicated that their image analysis approach was difficult to generalize to different size plates since the algorithm used was specific to the image size and resolution [25].

One of the simplest zebrafish toxicity screening assays is based on optical imaging and evaluating the general morphology and developmental status of zebrafish embryos and larvae (identified by different phenotypes) [33]. Toxicity of eNMs can be inferred from the phenotypes of treated zebrafish embryos. For example, the “dead” phenotype indicates a highly toxic effect, “unhatched” (with the embryo staying alive) indicative of interference in embryo development and a “hatched” phenotype signifying little toxicity over the course of the assay. In addition to providing qualitative toxicological analysis, phenotypes can be readily used to construct scores or ranking of mortality (i.e., rate of embryo death), hatching failure or success rates [26]. Within the context of eNM toxicity, it has been reported that ZnO and Cu nanoparticles can retard embryo hatching even leading to lethalty [21], [34], [35], quantum dots capable of hatching interference [4], and exposure to silver nanoparticles leading to a high rate of zebrafish embryo mortality [4], [24]. Given the emerging interest in the large scale implementation of zebrafish HTS to evaluate eNM toxicity, it is essential to develop a rapid and automated analysis of captured zebrafish images for phenotype recognition. This is a particular challenge for grayscale images [33], and where capture images can be blurred by noise arising from nanoparticle deposits and zebrafish chorion fragments.

In the present work, a new image recognition system is proposed to enable rapid automatic phenotype identification of zebrafish embryos exposed to eNMs without fluorescence based imaging. In the system, a machine learning model for phenotype recognition is proposed, instead of relying on visual inspection by a trained eye. The recognition ability of the current approach is demonstrated for three basic embryonic zebrafish phenotypes (i.e., hatched, unhatched, and dead embryos) based on 1153 training images and a stress test set of 96 images of low quality (not used for model training), both obtained in a toxicity screening of eNM treated zebrafish embryos.

## Methods

### Problem Formulation and Zebrafish Images

The *in vivo* HTS of eNM toxicity using a zebrafish embryo phenotype-based assay comprises of automated embryo plating, imaging, and phenotype identification. In the present work, a phenotype recognition system was developed based on images obtained from a previously published study on HTS zebrafish toxicity screening of eNMs where the details of the experimental protocol and automated imaging are provided [33]. Briefly, during automatic plating, healthy zebrafish embryos are selected, one embryo at a time, and placed into HTS plates, with each well containing the dispersed eNMs over a range of specific concentrations. After a prescribed exposure time, the automatic imaging system takes well-by-well images to reveal the development status of the zebrafish embryos. For the HTS system (which is described in [33]), three basic embryonic phenotypes (hatched, unhatched, and dead) were used as the toxicity indicator of eNMs. These are the most commonly used phenotypes in zebrafish studies of eNMs toxicity [33]. Although it is possible to define more subtle (or intermediate) sub-phenotypes, especially for the hatched larvae, the biological significance of such sub-phenotypes are yet not well understood, especially within the context of nano-toxicity. Moreover, it is noted that in order to capture sub-phenotypes, a significant degree of human intervention is required to manipulate the embryos/larvae positioning/alignment (e.g., by first anesthetizing zebrafishes) for detection in two-dimensional images [36]. Such an approach requires significant effort and is not suitable for high throughput screening of large numbers of eNMs over a wider range of concentrations. On the other hand, high throughput screening that makes use of automated imaging that resolves the image orientation/positioning challenge (without the need for manual intervention) is feasible and can be accelerated, as shown in the present work. This can be accomplished through automated image recognition of the three basic phenotypes that are generally accepted as reasonable indicators of *in vivo* toxicity [33].

**Figure 1:**
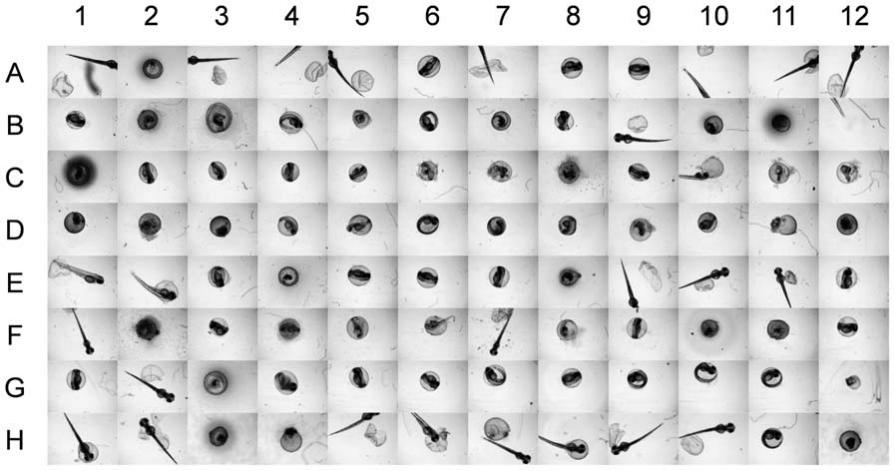
Images captured from a 96-well plate of zebrafish embryos. The embryos were treated with silver nanoparticles of concentration up to 15 mg·L^−1^. Examples of different phenotypes are: A1, E1, F1, H1→hatched embryos; B1, G1, C1→unhatched embryos; C1, D1, H12→dead embryos. C8, F2, and F7 illustrate images with significant deposits of eNMs. Images with chorion (eggshell) fragments are shown in A5, H7, and H8. Examples of zebrafish that are not completely captured by the imaging system (i.e., only a central portion of the well is imaged) are shown in A4, A7, and A10.

Examples of a set of images depicting the three phenotypes: hatched (e.g., A1, E1, F1, and H1), unhatched (e.g., B1, G1, and C1), and dead (e.g., C1, D1, and H12) embryonic phenotypes is shown in Figure 1. The captured images include some that are of low quality due to interference by eNMs deposits (e.g., C8, F2, and F7) and/or chorion (eggshell) fragments (e.g., A5, H7, and H8). It is also noted that because the images only cover the center ∼32% of the surface area of each well, a number of the images of the hatched larvae, did not include the whole organism (e.g., A4, A7, and A10). Subsequent to image capture, image analysis is carried out to identify the embryonic phenotypes, which represent different eNM toxicity levels.

Heuristic approach and machine learning are the two major approaches to automate image analysis for phenotype identification (without user intervention). However, as is evident from Figure 1, it would be difficult to construct a simple heuristic rule that can capture the subtle difference between unhatched and dead embryos, particularly in the presence of significant particle deposition. Furthermore, considering the complexity of the current images (the embryos/larvae position/orientation varies across images), a heuristic approach [25] may result in proliferated rules and provide results of less generalization [37]. On the other hand, if a sufficiently large dataset of images is available, then a machine learning approach can be effective for developing a phenotype recognition model of good generalization capability. In such an approach, a classification model was trained to recognize the three phenotypes initially identified based on an expert eye classification. The developed model is then used for automated phenotype recognition in subsequent HTS studies with the specific system for which the model was developed. The phenotype recognition model was developed using a set of images generated in a high throughput screening (HTS) assay that involved embryo exposure to CuO, ZnO, NiO, Co_3_O_4_, and silver nanoparticles (primary size range of 10 nm–40 nm and concentration range of 0.1–200 mg·L^−1^) in parallel with control wells (i.e., unexposed embryos). A total number of 1488 TIFF images (16-bit grayscale and 696×520 pixel resolution) were captured from 16 96-well plates (with one of the plates only half populated) 72 hr after initial exposure. These images were converted into common 8-bit grayscale JPEG format for ease of subsequent image processing. Initial image inspection revealed that 194 images were unsuitable for model development due to either extremely poor quality (including blurriness introduced by particle deposition) or well miss-plating (i.e., containing no embryo or more than one embryo). Zebrafish edema was observed in additional 45 images and these were also removed from the training set since only the three basic phenotypes (i.e., hatched, unhatched and dead) were included in the present classification model. The remaining 1249 images were then processed using the Caliph & Emir image analysis software [38] to detect and enumerate the number of edges in each image (images are accessible at http://nanoinfo.cein.ucla.edu/public/data/zim.zip). Visual inspection of the image set revealed that zebrafish images with less than about 170 edges were generally of good quality. However, blurry images of wells with high nanoparticle concentration were determined to have more than 170 edges. Accordingly, 96 of the remaining lower quality images were set aside for a subsequent stress test (i.e., for external validation) of the developed classification model. The final filtered set of 1153 images of good quality were then selected for expert phenotyping (i.e., by visual inspection) that identified 528, 327, and 298 of the images as those of hatched, unhatched, and dead embryos, respectively. This labeled set of images was used for model training and cross-validation for the above three zebrafish phenotypes.

### Automated Phenotype Recognition

**Figure 2:**
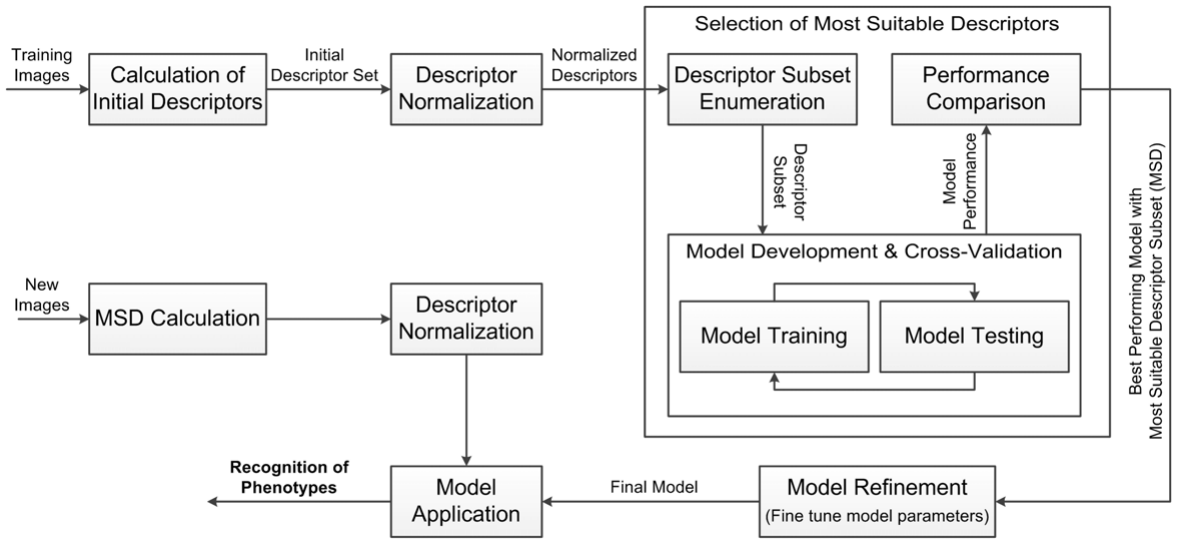
Workflow of phenotype recognition model development for *in vivo* HTS toxicity assay using zebrafish embryos.

The development of automated phenotype recognition for zebrafish embryo HTS followed the workflow depicted in Figure 2. First, an initial set of image descriptors [39] were calculated to construct a compact representation to characterize raw image content information. Following normalization of the initial descriptors, the most suitable descriptors were identified via model development and cross-validation with different descriptor combinations (i.e., descriptor selection [40]). Subsequently, the best performing model was attained by fine tuning model parameters to further improve recognition accuracy. Phenotyping of new images is then accomplished with the final model post calculation and normalization of the pertinent (i.e., most suitable) image descriptors. The above approach is detailed in the subsequent sections.

### Image Descriptors

An initial set of six color and texture descriptors were calculated and evaluated for the development of zebrafish phenotype recognition model. Three of the descriptors are the standard MPEG-7 (a multimedia content description standard) descriptors [41], [42] including (a) Local Edge Histogram Descriptor (LEHD), (b) Color Layout Descriptor (CLD), and (c) Scalable Color Descriptor (SCD). These descriptors provide compact image representations suitable for image-to-image matching and enable retrieval of images with similar semantics (e.g. the zebrafish phenotypes). Three additional texture and color descriptors were constructed in order to increase the discriminative ability for the zebrafish images, namely: (a) Global and Semi-global Edge Histogram Descriptor (GSEHD) [43], (b) Representative Color Descriptor (RCD), and (c) Color Histogram Descriptor (CHD). It is noted that within the context of the present work color descriptors describe grayscale information of the captured bright-field images. The determination of the above three constructed descriptors is described below along with a brief description of the MPEG-7 descriptors that were calculated using the Caliph & Emir software [38].

**Figure 3:**
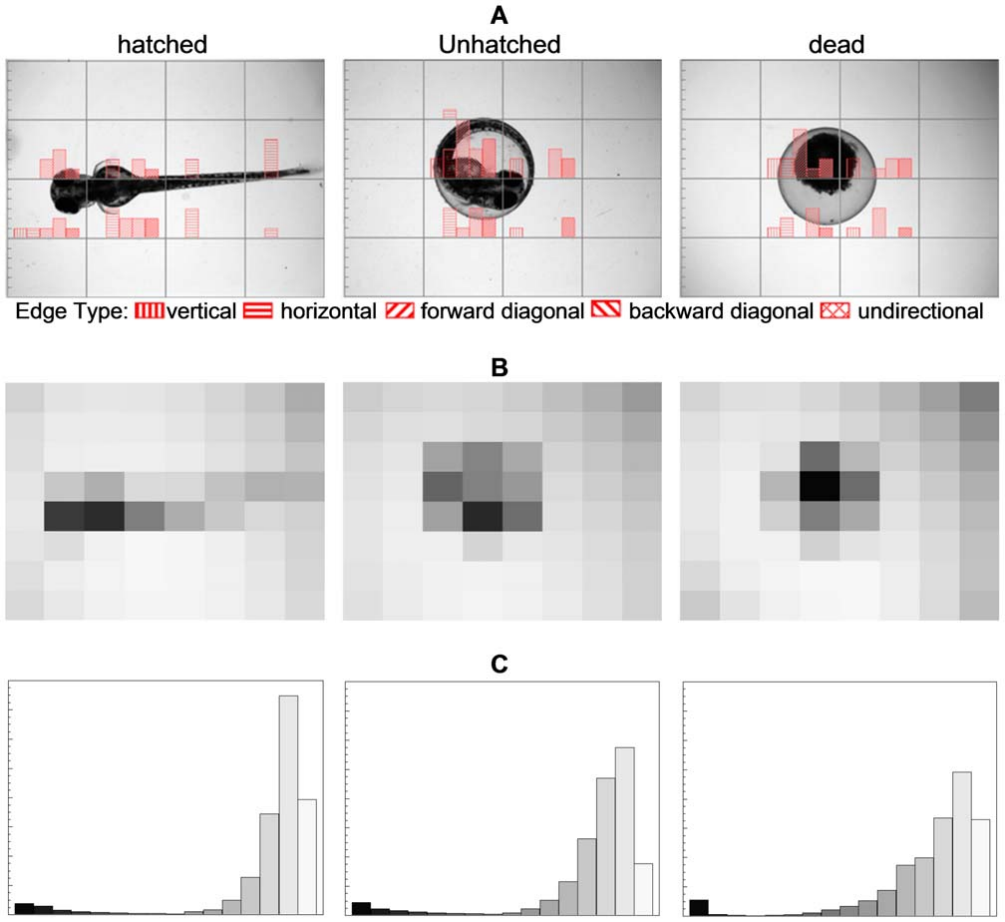
Examples of the three phenotypes and their corresponding image descriptors. (a). Local Edge Histogram for each of 4×4 image blocks (*y* axis of each of the 4×4 image blocks is from 0 to 6. (b). Representative Color (i.e., the average color (grayscale) for each of the 4×4 image blocks). (c). Color Histogram (*x* axis is the graryscale that ranges from 0 to 255; *y* axis is from 0 to 16×104 identifying the number of pixels which grayscale are within the bin range).

**Figure 4:**
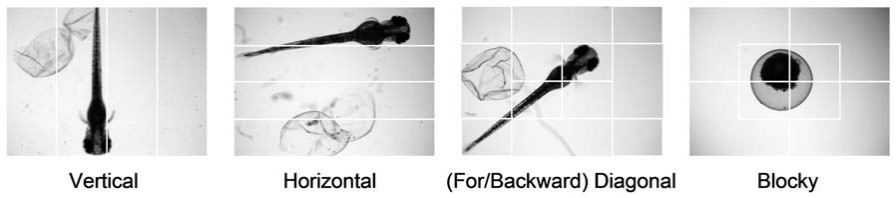
Sub-image segments for defining Semi-global edge histograms. The segmentations are corresponding to the typical layouts of zebrafish embryos of the constructed GSEHD descriptor.

The LEHD [42] descriptor provides texture information in terms of the spatial distribution of five types of edges, i.e., vertical, horizontal, forward diagonal, backward diagonal, and undirectional edge. LEHD comprises 80 ( = 16×5) histogram bins (i.e., a vectorial descriptor of dimension 80) corresponding to the distribution of the five different edge types over 4×4 non-overlapping image blocks of equal size (i.e., the image is divided into 4×4 equal blocks). Examples of LEHD are given in Figure 3(a) for three typical zebrafish images corresponding to each of the three phenotypes analyzed. The 80-bin LEHD specified by MPEG-7 only provides local texture semantics represented by the edge distribution and by itself may be insufficient to yield efficient image-to-image matching. Therefore, the Global and Semi-global Edge Histogram Descriptors (GSEHD) [43] were constructed by aggregating (i.e., adding) the block histograms of the entire image and five sub-image groups comprised by 4 blocks (corresponding to the typical layouts of zebrafish embryos, Figure 4). Accordingly, the generated GSEHD vectorial descriptor comprised of 80 histogram bins.

The CLD [42] descriptor captured the local spatial distribution of color in the zebrafish images by using the coefficients of the 8×8 Discrete Cosine Transformation (DCT) [44] on the representative color (the average color of equally partitioned 8×8 non-overlapping sub-images) in YCbCr color space [42]. In the present work, 15 low frequency coefficients [45] of the DCT for the Y component (i.e., a vectorial descriptor of dimension 15), which is essentially the grayscale of an image, were used in order to keep the major color layout of the zebrafish images. The coefficients of Cb and Cr components were not used since for a grayscale image they are constant and non-informative. The spatial color distribution can be also informed from the representative color before the DCT transformation. Therefore, the Representative Color Descriptor (RCD) comprised of 64 representative colors (i.e., a vectorial descriptor of dimensional 64, see Figure 3(b) for example) was also evaluated in the present work.

SCD is a Haar transform encoded color histogram in HSV color space [42], which characterizes an image by the global color distribution. The standard SCD comprises of 256 coefficients but for grayscale images only 8 (i.e., a vectorial descriptor of dimension 8, corresponding to 4 levels of the V color component, which again is corresponding to the grayscale) are non-constant. In order to improve the SCD resolution, a 16-bin Color Histogram Descriptor (CHD) was constructed as illustrated in Figure 3(c) for the three zebrafish images.

The GSEHD, SCD, and CHD are global descriptors capturing overall information about the images. These descriptors also support translation/rotation-invariant image-to-image matching and thus are especially suitable for phenotyping since zebrafish and embryos may appear at any location and orientation within the image area. However, the main issue of using global descriptors alone in image recognition occurs when images of different content (i.e., semantics) having similar global color and texture information In such a situation, the addition of descriptors such as LEHD, CLD, and RCD provide local (spatial) color and texture information that can increase phenotype discriminative ability.

The above six vectorial image descriptors contain 263 characteristics (i.e., vector components, 103 for the three standard MPEG-7 descriptors and 160 for the three constructed ones) of significantly different dynamic ranges (e.g., The LEHD is within [0, 7] while CHD can rise up to 16×10^4^). In order to prevent miss-weighing the importance of the descriptors that might be contingent upon their dynamic range, all the 263 characteristics were normalized using Z-score [46] (defined by *z* = (*c−μ*)/*σ* with *μ* and *σ* denoting the sample mean and standard deviation of a descriptor component *c*) for the following descriptor selection and model development. It is noted that for the training set the above normalization resulted in the descriptor components each having a zero mean and standard deviation of unity.

### Descriptor Selection and Model Development

Descriptor selection was conducted to identify (vectorial) descriptors of good phenotype discriminative ability. The process of descriptor selection and model development were integrated into a wrapper descriptor selection scheme [40]. The discriminative ability of each possible descriptor subset (for six descriptors there are 2^6^ = 64 such subsets) was assessed by the 10-fold cross-validation recognition accuracy [47] of the corresponding image classification (recognition) model. The 10-fold cross-validation is a recursive technique for estimating model performance based on partitioning a data set into ten mutually exclusive subsets, with nine subsets used for training and one for validation. The process is repeated for each of the 10 subsets in order to obtain the averaged model performance [47]. As a result, the best-performing model and its underlying descriptors were identified simultaneously. Finally, the current best performing model was fine-tuned in order to further improve its recognition accuracy.

**Figure 5:**
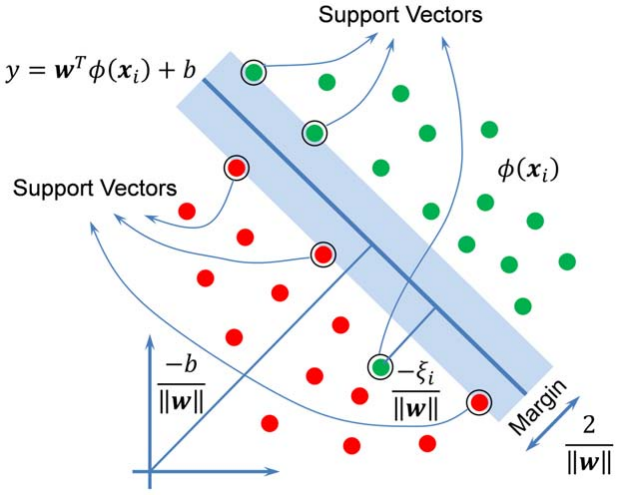
Geometric description of SVM for a binary classification problem.

The classification model was developed based on the Support Vector Machine (SVM [48], [49]) which is depicted geometrically in Figure 5 for a two-class classification problem. For the present ternary classification problem the LibSVM [50] package was used, utilizing the “one-against-one” approach [50], [51] to decompose a *k*-class classification into *k*(*k*−1)/2 binary classification problems.

In the SVM approach, data in the original input space (i.e., the space defined by the set of image descriptors) are (non-linearly) mapped onto a higher dimensional space (*ϕ*(***x*** *_i_*)) so that they are more likely to be linearly separable. Subsequently, an optimal classifier (***w*** *^T^ϕ*(***x***)+*b*) is found by the SVM that maximizes the margin (2/∥***w***∥, Figure 5) between the two classes and minimizes overall training error (Σ*ξ_i_*, Figure 5). Mathematically, the SVM is formulated as the following optimization problem:
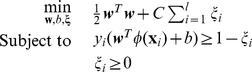
(1)In this formulation, (***x*** *_i_*, *y_i_*), *i* = 1, …, *l*, denote the training data, where ***x*** *_i_* is an input sample and *y_i_* ∈ {−1, 1} is its class label, and *ϕ*(·) is the function that maps the input data onto a higher dimensional space. The mapping can be implicitly defined by a kernel function which enables solving the nonlinear optimization problem linearly in a kernel space. In the present work, the Gaussian kernel [52] was adopted (eq. 2),
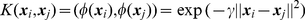
(2)The SVM with a Gaussian kernel involves two adjustable model parameters (*C*, *γ*) which were determined based on a heuristic “grid-search” [52] that was conducted among *C* ∈ {2^−5^, 2^−3^, …, 2^15^} and *γ* ∈ {2^−15^, 2^−13^, …, 2^3^} with 10-fold cross-validation [47]. The best classification accuracy for the different models was then used to index the discriminative ability of the image descriptors. After the descriptor subset of the best discriminative ability was identified, the smallest grid covering the best (*C*, *γ*) was further divided into a 30×30 sub-grid of equal size units. An additional “grid-search” was then conducted on this refined grid to fine-tune (*C*, *γ*) and further improve the classification accuracy of the best performing model.

## Results and Discussion

The six image descriptors (i.e., LEHD, GSEHD, CHD, SCD, RCD, and CLD) were evaluated via 10-fold cross-validation for the SVM developed with all possible descriptor combinations (i.e., subsets). The classification accuracy (i.e., phenotype recognition accuracy) is summarized in Table 1 for each descriptor subset.

**Table 1:** Discriminative ability of all the descriptor subsets indexed by the 10-fold cross-validated SVM classification accuracy.

Subset^a^	Acc^b^ (%)	Subset	Acc (%)	Subset	Acc (%)	Subset	Acc (%)
000000	N/A	001111	97.05±1.30	011111	96.96±1.56	110000	90.11±2.25
000001	94.80±2.12	010000	89.34±3.13	100000	90.20±1.98	110001	93.41±2.15
000010	95.49±3.08	010001	94.45±2.42	100001	93.67±2.35	110010	94.71±2.77
000011	95.66±2.27	010010	95.66±2.32	100010	94.19±2.82	110011	95.06±2.84
000100	65.31±5.01	010011	95.67±3.35	100011	94.62±2.59	110100	92.28±1.74
000101	95.49±2.39	010100	93.06±2.28	100100	91.85±1.97	110101	94.02±2.02
000110	96.18±1.66	010101	95.93±2.38	100101	95.14±2.16	110110	95.40±2.12
000111	96.27±1.90	010110	95.84±2.56	100110	95.32±2.30	110111	96.01±1.90
001000	84.04±2.41	010111	96.19±2.23	100111	95.84±2.04	111000	93.32±3.02
001001	96.79±1.51	011000	94.62±2.35	101000	93.84±2.87	111001	95.40±1.84
001010	96.96±1.12	011001	96.53±1.82	101001	96.10±2.27	111010	96.18±2.43
001011	**97.14±1.03**	011010	96.53±1.45	101010	96.36±1.54	111011	96.62±1.52
001100	83.69±2.93	011011	96.88±1.51	101011	96.44±1.95	111100	93.49±2.38
001101	96.18±1.87	011100	95.14±2.02	101100	94.62±2.80	111101	95.49±1.93
001101	96.18±1.42	011101	96.70±1.68	101101	96.44±1.80	111110	96.18±2.27
001110	96.79±1.29	011110	96.88±1.35	101111	96.53±1.82	111111	96.62±1.52

a The feature subsets are coded by binary vectors with “1” indicating the presence of a feature group while 0 denoting its absence. For example, feature subset {LEHD, GSEHD, CHD, SCD, RCD, CLD} is coded by “111111”.

b Acc: the average classification accuracy (± standard deviation) obtained via 10-fold cross-validation for the developed SVM.

Among the six image descriptors, SVM models based on either SCD or CHD as single descriptors (i.e., corresponding to subsets “000100” and “001000”) performed with a relatively low phenotype recognition accuracy of 65.3±5.01% and 84.04±2.41%, respectively (Table 1). Somewhat increased classification accuracy of 90.20±1.98% and 89.34±3.13% was obtained for single descriptor models based on the LEHD (“100000”) and GSEHD (“010000”), respectively (Table 1). It is noted that a model containing both of the above two edge histogram descriptors (subset “110000”) demonstrated limited image classification accuracy of 90.11±2.25% (Table 1); the above behavior is attributed to the possible distortion of the edge histograms when a hatched embryo eggshell remains in the imaged area or when there is excessive deposition of nanoparticle. Moreover, unhatched and dead embryos are similar in edge histograms (e.g., Figure 3(a)) and thus are difficult to discriminate solely by edge histograms. The use of RCD or CLD in single descriptor based models ameliorated the above deficiencies by averaging the color in the image which was partitioned into 8×8 blocks (e.g., Figure 3(b)). This approach resulted in superior SVM models with classification accuracy of 95.49±3.08% and 94.80±2.12% (Table 1) for the RCD and CLD based models (i.e., corresponding to subsets “000010” and “000001”), respectively.

The SVM classification model developed with the three constructed descriptors (GSEHD, CHD, and RCD, i.e., subset “011010”) demonstrated better classification with reduced standard deviation (96.53±1.45%). A model based the three standard MPEG-7 descriptors (LEHD, SCD, and CLD; i.e., subset “100101”) yielded somewhat lower classification accuracy of 95.14±2.16%. The improved accuracy with the constructed descriptors (Subset “011010”) can be attributed to a greater discriminative ability with their total of 160 vectorial descriptor components relative to 103 components of the three standard MPEG-7 descriptors. Incorporation of information regarding different granularities (i.e., different resolution levels) by including all of the six descriptor sets (i.e., Subset “111111”; Table 1) improved the classification accuracy to 96.62±1.52%. Out of the 64 possible descriptor combinations there were 26 subsets that resulted in SVM classifiers with accuracy higher than 96% with two of the models (i.e., with descriptors {CHD, RCD, CLD} and {CHD, SCD, RCD, CLD}; corresponding to Subsets “001011” and “001111” in Table 1) with classification accuracy above 97%. The SVM model based on the {CHD, RCD, CLD} descriptor subset demonstrated the best classification accuracy of 97.14±1.03%. It is noted that a slightly lower performance 97.05±1.30% was obtained upon the addition of the SCD descriptor to the best performing three-descriptor model. The lower performance of the {CHD, SCD, RCD, CLD} descriptor set is possibly due to the fact that the SCD discretizes V component of the HSV color space (which is corresponding to the grayscale of an image) only into four levels (bins) and thus its use introduces noise into the model when it is used along with the higher resolution CHD descriptor which contains sixteen grayscale bins.

As an alternative to the SVM models, the *k*-Nearest Neighbors (*k*-NN) [53] algorithm was also evaluated. In the present approach, the parameter *k* was set to its typical default value of ten [53]. The best performing *k*-NN models were with the descriptor sets {CHD, CLD}, {CHD, SCD, CLD}, and {CHD, RCD, CLD} which provided classification accuracies of 91.93±2.53%, 91.50±2.48%, and 91.41±2.63%, respectively. It is interesting to note that although the *k*-NN models were of lower accuracy relative to the SVM based models, {CHD, RCD, CLD} which was the most suitable descriptor subset for the SVM model was among the three best performing subsets (all within a recognition accuracy of 91%–92%) for the *k*-NN based models.

The best performing SVM model (i.e., the SVM model developed with the {CHD, RCD, CLD} descriptor set and model parameters *C* = 2^3^ and *γ* = 2^−7^ obtained via the initial grid search) was further improved via a refined grid search to arrive at the optimal model parameters searched over the range of *C* ∈ [2^1^, 2^5^] and *γ* ∈ [2^−9^, 2^−5^]. The optimal *C* and *γ* parameters were found to be 5 and 2^−7^, respectively, resulting in SVM model classification accuracy that increased to 97.40±0.95%. The detailed classification performance of the above model is presented in Table 2 in the format of a confusion matrix. In this matrix class recall (i.e., percentage of the samples in a given class that are correctly identified) represents the system error of the developed SVM classifier for auto-phenotyping when eNM toxicity is measured by the rates of hatched, unhatched, and dead embryos (i.e., mortality rate, hatching rate). The SVM classifier performs with high class recalls for the hatched and unhatched phenotypes (99.24% and 98.17%, respectively) with lower recall (93.29%) for the “dead” phenotype. It is noted that the “false-positive” rate for each phenotypes can be quantified as: 100% - class precision. For example, the “false-positive” rate of the “dead” phenotype is 0.71% ( = 100%−99.29%) which indicates that two out of the 280 images predicted as belonging to the “dead” phenotype were misclassified although they were actually “unhatched” embryos. Overall, however, the false-positive rate with the best performing SVM classifier was less than 5% for the three phenotypes.

**Table 2:** Performance of the SVM phenotype recognition model in 10-fold cross-validation^a^.

	true hatched	true unhatched	true dead	class precision^b^
**pred. hatched**	524	4	8	97.76%
**pred. unhatched**	4	321	12	95.25%
**pred. dead**	0	2	278	99.29%
**class recall** c	99.24%	98.17%	93.29%	

a The overall recognition accuracy is 97.40±0.95%.

b The class precision is the percentage of correct classified samples in a predicted class. For example the precision of the (predicted) hatched class is given by 524/(524+4+8) = 97.76%.

c The class recall is the proportion of the samples in the class that were correctly identified. For example the recall of (true) hatched class is 524/(524+4+0) = 99.24%.

**Figure 6:**
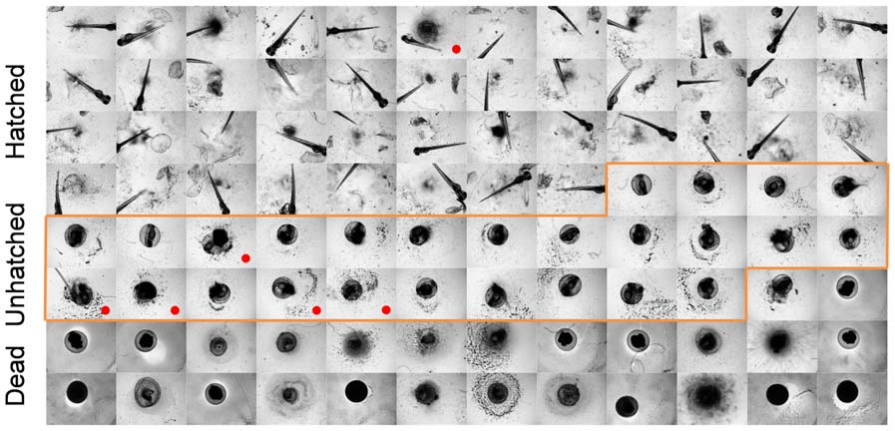
Stress test set composed by 96 images of low quality. Red dot identifies the images that were misclassified (all as “dead” phenotype)).

The recognition ability of the final SVM classifier was also intensively assessed (via the recognition phase for new images depicted in Figure 2) using the stress test with 96 low quality images “unseen” by the model (i.e., these images were not used to train the model). The phenotype classification performance for this “stress” test is given in Table 3 and the misclassified images are tagged with red dot in Figure 6.

**Table 3:** Performance of the SVM phenotype recognition model in the stress test^a^.

	true hatched	true unhatched	true dead	class precision
**pred. hatched**	43	0	0	100.00%
**pred. unhatched**	0	22	0	100.00%
**pred. dead**	1	5	25	80.65%
**class recall**	97.73%	81.48%	100.00%	

a The overall recognition accuracy is 93.75%.

The classifier performance with the lower quality stress set images (Table 3) was with recognition accuracy lower by 3.65% relative to that which was obtained win the 10-fold cross-validation test (Table 1). As indicated in Figure 6, six of the 96 stress test images were misclassified (all as a “dead” phenotype), likely due to significant nanoparticle deposits that are seen as large dark spots (about the unhatched embryos) that are confused with dead embryos.

**Figure 7:**
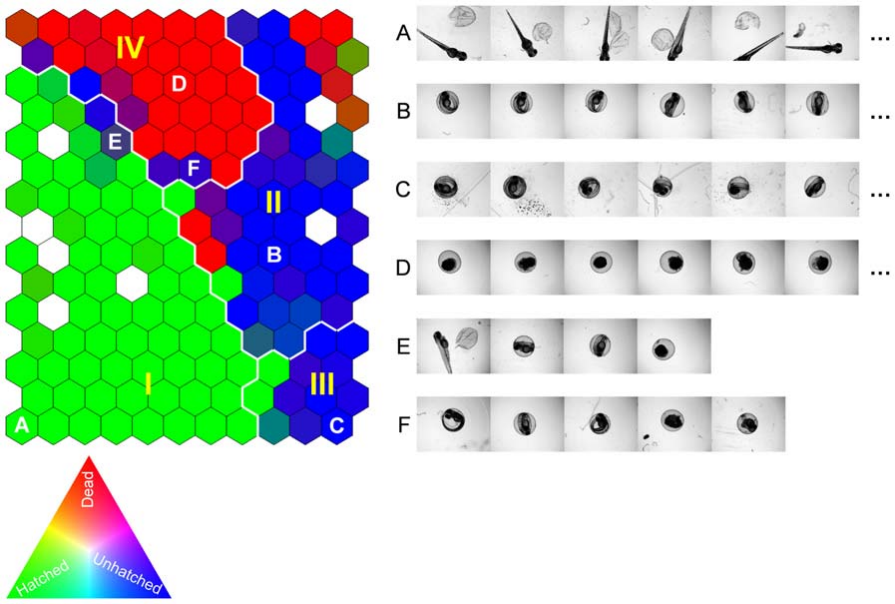
Self-Organizing Map (SOM) of the training image set described by the three selected image descriptors. Similar images are organized as neighbors on the map and clusters I–IV are the four primary clusters identified by SOM analysis. The SOM cells are colored by [R, G, B] = [*N_dead_*, *N_hatched_*, *N_unhatched_*]/*N*, where *N_dead_*, *N_hatched_*, and *N_unhatched_* identify the number of dead, hatched, unhatched zebrafish embryos grouped into a given cell, respectively, and *N* = *N_dead_*+*N_hatched_*+*N_unhatched_*. Accordingly, homogeneous SOM cells of hatched, unhatched, and dead zebrafish embryos (e.g., A, B & C, and D) are colored by pure green, blue, and red, respectively. White colored cells are empty cells (no images grouped in the cells). Examples of images grouped in the same SOM cells are given in the corresponding image rows to the right of the SOM.

Finally, in order to demonstrate the intrinsic ability of the optimal descriptor subset {CHD, RCD, CLD} for assessing similarity/dissimilarity of the zebrafish images, Self-Organizing Map (SOM) [54], [55] analysis was conducted with the training image set using the above descriptors but without the phenotype information. In this unsupervised SOM analysis similar images (with respect to the three selected descriptors) were organized in a two-dimensional discretized map on which four primary clusters were identified (clusters I–IV, Figure 7). In the representation of Figure 7, each SOM cell was colored with RGB scale proportional to the number of dead, hatched, and unhatched zebrafish embryo images grouped in the cell. As a result, homogeneous cells that contain images of only one (in each cell) of the hatched, unhatched or dead phenotypes are colored green, blue, and red, respectively. Heterogeneous SOM cells which contain images of different phenotypes are identified with a mixed color of RGB components that is proportional to the number of dead, hatched, and unhatched zebrafish embryo images grouped in the cell. The resulting pictorial mapping in Figure 7 indicates that the majorities of the hatched and dead phenotypes are grouped into clusters I and IV, respectively. While clusters II and III comprise mainly of SOM cells representing the unhatched phenotype and thus are essentially very similar in their representation (i.e., can be considered as a single metacluster). It is noted that most of the heterogeneous cells (i.e., containing a mix of phenotypes) are located at the boundaries of the clusters, representing images that are difficult to differentiate with the three descriptors.

In order to further explore the merit of SOM clustering of the images on the basis of the descriptors alone, one can explore the image content of each SOM cell. As an illustration, a selection of homogeneous SOM cells, identified as A, B & C, and D, are provided in Figure 7 for hatched, unhatched, and dead phenotypes, respectively. Cells A and B & C consist images of hatched and unhatched zebrafish embryos of different orientations (image rows A and B & C, Figure 7); this demonstrate that the selected descriptors are sensitive mainly to image semantics (i.e., zebrafish phenotype), irrespective of (internal/external) embryo orientation. The images grouped in cell A also suggest that the selected descriptors are not sensitive to noise arising from eggshell fragments and nanoparticle deposits. In contrast to cells A–D, cells E and F are examples of heterogeneous SOM cells that group images of different phenotypes (image rows E and F, Figure 7) that are difficult to discriminate by an unsupervised approach (i.e., without training a model in a supervised course with the additional phenotype information). Finally, in order to quantify the cluster quality, similar to the confusion matrices (Table 2 and Table 3) for classification, the class precision and recall were calculated and given in Table 4 with cluster II and III considered as a single metacluster.

**Table 4:** Quality of the clusters identified by SOM analysis^a^.

	hatched	unhatched	dead	class precision
**cluster I**	492	14	10	95.35%
**cluster II** b	21	229	73	72.37%
**cluster III**	13	67	6	
**cluster IV**	2	17	209	91.67%
**class recall**	93.18%	90.52%	70.13%	

a Average cluster quality is 86.47%.

b Cluster II and III considered as a combined cluster.

The SOM clusters grouped the images of the same phenotypes with a reasonable accuracy of 86.47% without utilizing the phenotype information. This demonstrates that the selected descriptors provide a suitable level of image description that is not sensitive to embryo orientation but is highly sensitive to the zebrafish phenotype. This suggests that, the presently selected descriptor subset may assist, via SOM analysis, in the interpretation of zebrafish embryo based *in vivo* HTS studies by providing preliminary identification of the number of different phenotypes that may be present in the image set.

### Conclusions

An automatic phenotype recognition system was developed in order to facilitate HTS zebrafish toxicity screening of eNMs in which the developmental response of zebrafish embryos was classified by three basic phenotypes (i.e., hatched, unhatched and dead) based on analysis of captured optical images. Accordingly, a support vector machine based phenotype recognition model was developed with a set of three image descriptors (i.e., color histogram, representative color, and color layout). These selected descriptors were identified from an initial pool of six vectorial image descriptors providing information regarding color and texture characteristics. The best phenotype recognition model performed with an average classification accuracy of 97.40±0.95% in a 10-fold cross-validation and 93.75% classification accuracy for a stress test with zebrafish images of low quality. The performance and robustness of the current automatic phenotype recognition system is encouraging and suggest its practical use for high throughput zebrafish-based toxicity testing. Moreover, irrespective of the materials (e.g., nanoparticles, chemicals, etc.) to be tested, the present methodology for developing a phenotype recognition system should be applicable, without a loss of generality, to other nanoparticle systems. Finally, although the present recognition model was demonstrated for only three basic embryonic phenotypes, with a sufficiently large and diverse dataset, the modeling approach can be extended to enable identification of more subtle phenotypes.
